# Supplementary material for: ezRAD: a simplified method for genomic genotyping in non-model organisms
Source: PeerJ. 2013 Nov 19;1:e203. doi: 10.7717/peerj.203 (PMC3840413; doi:10.7717/peerj.203)
Supplement: File S1 [file peerj-01-203-s001.pdf]

ezRAD Modifications to Illumina TruSeq DNA Kit Protocol

Modified from Ethan Ford's TruSeq RNA Kit protocol

http://ethanomics.files.wordpress.com/2012/02/truseq_rna_kit_protocol_third2.pdf

Perform End Repair

1. Use 13.3 µl of End Repair Mix instead of 40 µl.
2. Add 53.3 µl of Ampure XP Beads instead of 160 µl.
3. Place on magnetic rack for 5 minutes instead of 15 minutes.
4. Add 10 µl Resuspension Buffer instead 17.5 µl.
5. Incubate at room temperature for 5 minutes instead of 2 minutes.
6. Transfer 9 µl of the supernatant to a new 0.2 ml PCR tube instead of 15 µl.

Adenylate 3' Ends

1. Add 4.17 µl A-Tailing Mix to sample instead of 12.5 µl.

Ligate Adapters

1. Change recipe to : 0.83 µl DNA Ligase Mix, 0.83 µl Resuspension Buffer, 0.83 µl Adapter Index .
2. Add 1.67 µl Stop Ligase Mix instead of 5 µl.
3. Add 20 µl of AMPure XP beads instead of 42.5 µl.
4. Place on magnetic rack for 5 minutes instead of 2 minutes.
5. Add 27 µl Resuspension Buffer instead of 52.5 µl.
6. Place in magnetic rack for 5 minutes instead of 2 minutes.
7. Transfer 26 µl of the supernatant to a new 0.2 ml PCR tube instead of 50 µl.
8. Add 26 µl of well-mixed AMPure XP beads instead of 50 µl.
9. Place on magnetic rack for 5 minutes instead of 2 minutes.

10. Add 25 µl Resuspension Buffer instead of 22.5 µl.

11. Place in magnetic rack for 5 minutes instead of 2 minutes.

12. Transfer 23 µl of the supernatant to a new 0.2 ml PCR tube.

Proceed directly to Enrich DNA Fragments

1. Modify PCR mix as follows: 10 µl of ligated DNA, 1.67 µl PCR Primer Cocktail, 8.33 µl PCR Master Mix.

2. Use 15-18 cycles of PCR instead of 10.

Return to Purify Ligation Products

1. Add 5 µl of 4X loading buffer to each sample and to ladder instead of 7 µl.
