# Supplementary material for: ezRAD: a simplified method for genomic genotyping in non-model organisms
Source: PeerJ. 2013 Nov 19;1:e203. doi: 10.7717/peerj.203 (PMC3840413; doi:10.7717/peerj.203)
Supplement: File S2 [file peerj-01-203-s002.pdf]

```bash
#!/bin/bash

#Script to automatically process X number samples, produce a reference assembly, map reads back to the assembly, and call SNPs

#Developed and written by Jonathan B. Puritz - jpuritz@gmail.com

#This pipeline depends on:

#Trim Galore! http://www.bioinformatics.babraham.ac.uk/projects/trim_galore/

#Cutadapt http://code.google.com/p/cutadapt/

#Rainbow http://sourceforge.net/projects/bio-rainbow/files/

#FASTX-Toolkit http://hannonlab.cshl.edu/fastx_toolkit/download.html

#BWA http://bio-bwa.sourceforge.net

#SAMtools http://samtools.sourceforge.net

#VarScan2 http://varscan.sourceforge.net

#VCFtools http://vcftools.sourceforge.net


###Make sure to update your $PATH settings and the paths to the jar and perl files###

###YOU MUST ENTER A MINIMUM ALLELE FREQUENCY WITH THIS SCRIPT#######

###IT CAN BE ESTIMATED BY 1 / (2 * THE NUMBER OF INDIVIDUALS)#############

###Rainbow (the assembler) needs this information to properly function####################

###Correct usage: sh ezRADpipline.sh <freq>#########################################

###Rename all FASTQ files to "Sample1.R1.fq" "Sample1.R2.fq"########################


###Begin Code###

#Simple way of keeping track of files

ls *.R1.fq > namelist

sed -i 's/.R1.fq//g' namelist
```

```
NAMES=( `cat "namelist" `)

mkdir assembly

#Trims raw files two different ways.

# First way removes any reads with substantial amounts of adapter, but does no quality trimming.  These reads are used for assembly and must be uniform lengths

# Second way removes adapters and does quality trimming.  These reads will be used for mapping.

for i in "${NAMES[@]}"

do

trim_galore --paired -q 0 --length 90 -a GATCGGAAGAGCACACGTCTGAACTCCAGTCACNNNNNNATATCGTATGCCGTCTTCTGCTTG -a2 GATCGGAAGAGCGTCGTGTAGGGAAAGAGTGTAGATCTCGGTGGTCGCCG --stringency 20 $i.R1.fq $i.R2.fq --output_dir ./assembly

trim_galore --paired -q 20 --length 20 -a GATCGGAAGAGCACACGTCTGAACTCCAGTCACNNNNNNATATCGTATGCCGTCTTCTGCTTG -a2 GATCGGAAGAGCGTCGTGTAGGGAAAGAGTGTAGATCTCGGTGGTCGCCG --stringency 10 $i.R1.fq $i.R2.fq

done

#Renaming trimmed files to simpler names

for i in "${NAMES[@]}"

do

mv $i.R1_val_1.fq $i.1.fq

mv $i.R2_val_2.fq $i.2.fq

done
```

```
###Assembly###

###These parameters could be further optimized for particular taxa

#First step concatenates reads into one forward and one reverse fastq file

cat ./assembly/*.R1_val_1.fq > forward

cat ./assembly/*.R2_val_2.fq > reverse

#Rainbow now clusters and assembles

rainbow cluster -1 forward  -2 reverse  > cat.rbcluster.out 2> log

rainbow div -i cat.rbcluster.out -o cat.rbdiv.out -f $1

rainbow merge -a -i cat.rbdiv.out -o cat.rbasm.out -N 1000

perl /path to/select_best_rbcontig.pl cat.rbasm.out > rainbow

#Renames contigs to sequential numbers for simplicity

fastx_renamer -n COUNT -i rainbow -o reference


##Mapping

#Use BWA to index reference

bwa0.7 index -a bwtsw reference


#Use BWA to map reads to reference.

###These parameters could be further optimized for particular taxa


for i in "${NAMES[@]}"

do

bwa0.7 mem reference $i.1.fq $i.2.fq -t 32 -a -T 10  > $i.sam

done
```

#Convert Sam to Bam and remove low quality, ambiguous mapping

for i in "${NAMES[@]}"

do

samtools view -bS -q15 $i.sam > $i.bam

samtools sort $i.bam $i

done

#Index reference for SAMtools

samtools faidx reference

##SNP Calling

###These parameters could be further optimized for a particular taxon

###Note that the this protocol keeps SAMTools BAQ calculation which may be too conservative for certain taxa. Also, VarScan can be futher optimized for individual and pooled samples.

#SAMtools performs local realignment around INDELs

samtools mpileup -D -f reference *.bam >mpileup

#VarScan calls all sites with at least 5X coverage, a variant frequency above 10%, and 95% probability of being a SNP

java -jar /path to/VarScan.v2.3.5.jar mpileup2snp mpileup --output-vcf --min-coverage 5 --strand-filter 0 --min-var-freq 0.1 --p-value 0.05 >SNPS.vcf

#VCFtools to filter raw SNPs and create a filtered vcf file (Final.recode.vcf) with SNPs that are present in every individual and that are not INDels

vcftools --vcf SNPS.vcf --geno 0.99 --out Final --recode --non-ref-af 0.001 --remove-indels

#VCFtools again to filter for SNPs that are present at an average of 10X coverage

```
vcftools --vcf Final.recode.vcf --out Final10X --recode --min-meanDP 10
```
