# Supplementary material for: ezRAD: a simplified method for genomic genotyping in non-model organisms
Source: PeerJ. 2013 Nov 19;1:e203. doi: 10.7717/peerj.203 (PMC3840413; doi:10.7717/peerj.203)
Supplement: File S3 [file peerj-01-203-s003.pdf]

We show here that ezRAD is able to reproducibly genotype the same sites in multiple individuals/libraries, which is particularly important for the purposes of SNP discovery and genotyping. We use a comparison of 8 individual libraries of a single fish each and 2 pooled libraries of 4 of those individuals and compare the results among the individual and pooled libraries.

We first used the 8 individual *P. arcatus* libraries to validate the performance of the ezRAD approach by comparing the number of shared SNPs among multiple libraries using three arbitrary coverage thresholds (10x, 20x, and 30x). In total, we genotyped 975 SNPs that were shared in all 8 individual libraries (at 10x mean coverage depth), 265 shared SNPs (at 20x coverage), and 113 shared SNPs (at 30x coverage). On average, 14% of all detected SNPs were shared and genotyped among all 8 libraries and ranged from 8% (10x) to 21% (30x) (Table S3-1). The effect of getting a higher shared SNP % by increasing the coverage threshold indicates that the method is targeting the same high-quality SNPs in each individual. In other words, the majority of SNPs that are not shared are of low coverage, and the SNPs that are shared across multiple individuals/libraries are scored consistently with higher coverage (Table S3-1). The impact of adding additional individuals (i.e., the reduction of shared SNPs among all individuals with a greater number of libraries) decreases beyond ~5-6 individuals, and the slope of the lines for 10x and greater coverage (Figure S3-1) indicates that adding additional individuals will not greatly reduce the total number of shared SNPs. These results validate that the ezRAD method can reproducibly genotype the same variable sites in multiple individuals.

In addition, we compared the number of shared SNPs in all 8 individual libraries to that of the 2 pooled libraries (of 4 individuals each), and found that of the 975 shared SNPs genotyped (at >10x mean coverage) in all 8 individual libraries, 635 (65%) were also genotyped in both pooled libraries. We suspect that this percentage is underestimated due to differences in lane coverage during sequencing: the 2 pooled libraries were allocated only 25% of the sequencing depth that was allocated to the 8 individual libraries (1/6 vs. 2/3 of a lane, see Table 1.). Thus, we expect that given equivalent sequencing coverage that the pooled libraries would identify most, if not all, of the SNPs shared among all 8 individual libraries, and at much lower cost as outlined in the text of the manuscript (Figure 1). Comparing the number of variable sites among the individual libraries (171,712) to those of the pooled libraries (20,512), illustrates the utility of the SNP calling which identified only 2,705 from the individual library (0.01 SNPs per contig) as compared to 10,221 from the pooled libraries (0.54 SNPs per contig) (Table 1). Overall, more SNPs of higher quality at lower cost were found from the pooled than the unpooled libraries.

Table S3-1. Percentage of SNPs shared amongst all individuals sequenced.

**Num. Indiv. Sequenced**	% of all SNPs Shared			**Average shared SNPs**
	**10x coverage**	**20x coverage**	**30x coverage**	
2	30%	38%	56%	41%
3	14%	26%	41%	27%
4	12%	21%	34%	22%
5	10%	18%	30%	20%
6	9%	17%	27%	18%
7	8%	15%	24%	16%
8	8%	14%	21%	14%

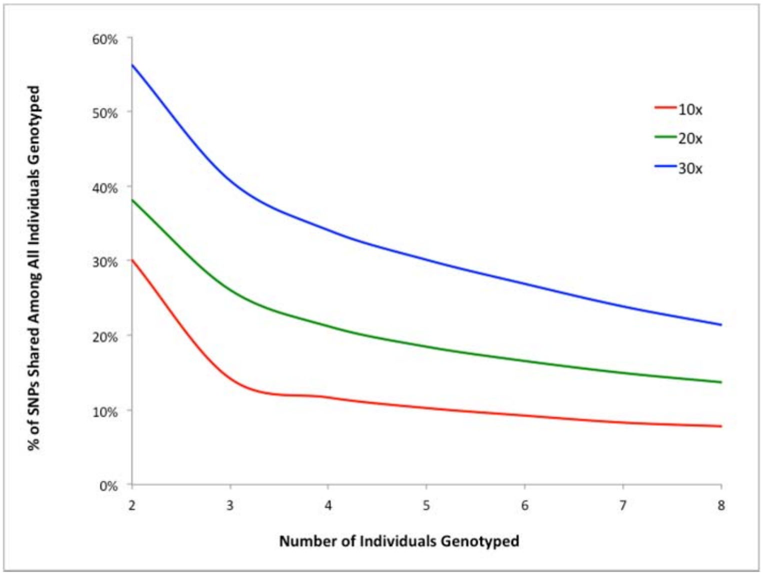

Figure S3-1. Line plot showing the percentage of SNPs that are shared among all individuals as additional individuals are sequenced. Data are presented using three thresholds of mean coverage depth at each site: 10x (red), 20x (green), and 30x (blue). These results validate the reliability of ezRAD to reproducibly genotype the same variable sites in multiple individual libraries: the % of SNPs shared among all 8 individuals ranges from 8-21% depending on coverage threshold used. The results highlight that the highest coverage SNPs tend to have higher rates of reproducibility among individual libraries than lower coverage SNPs; on average, the SNPs shared among all individuals tend to be shared with high coverage and reliability.
